# PDBalert: automatic, recurrent remote homology tracking and protein structure prediction

**DOI:** 10.1186/1472-6807-8-51

**Published:** 2008-11-25

**Authors:** Vatsal Agarwal, Michael Remmert, Andreas Biegert, Johannes Söding

**Affiliations:** 1 Department of Biotechnology, Indian Institute of Technology, Roorkee 247667, India; 2 Gene Center Munich and Center for Integrated Protein Science (CIPSM), Dept. of Chemistry and Biochemistry, Ludwig-Maximilians-Universtät München, Feodor-Lynen-Str. 25, 81377 Munich, Germany

## Abstract

**Background:**

During the last years, methods for remote homology detection have grown more and more sensitive and reliable. Automatic structure prediction servers relying on these methods can generate useful 3D models even below 20% sequence identity between the protein of interest and the known structure (template). When no homologs can be found in the protein structure database (PDB), the user would need to rerun the same search at regular intervals in order to make timely use of a template once it becomes available.

**Results:**

PDBalert is a web-based automatic system that sends an email alert as soon as a structure with homology to a protein in the user's watch list is released to the PDB database or appears among the sequences on hold. The mail contains links to the search results and to an automatically generated 3D homology model. The sequence search is performed with the same software as used by the very sensitive and reliable remote homology detection server HHpred, which is based on pairwise comparison of Hidden Markov models.

**Conclusion:**

PDBalert will accelerate the information flow from the PDB database to all those who can profit from the newly released protein structures for predicting the 3D structure or function of their proteins of interest.

## Background

With the advent of remote homology detection methods relying on the pairwise comparison of sequence profiles, automatic protein structure prediction has become reliable and sensitive enough to be of more general use[1]. For more than half of all proteins in representative genomes, at least one domain can be modelled with decent accuracy by fully automatic methods [[2]; J. Soeding, unpublished data]. When no template can be identified, the user will typically rely on keyword tracking services or regular manual checks of the PDB[3] to find out if a related structure has been released. But keyword searches will miss most of the useful templates, since paralogous proteins generally have different names while most will be sufficiently related to serve as templates for homology modelling or to generate hypotheses about possible functions.

Several freely available automatic systems have been developed to perform sequence searches periodically and to notify users about interesting hits. Earlier tools use BLAST[4] to search Swiss-Prot[5] or the non-redundant sequence database at the NCBI: Swiss-Shop[6], DBWatcher[7], BLAST Search Updater[8], and Sequence Alerting System[9]. FastAlert[10] uses FASTA[11] to search the Swiss-Prot, EMBL data library and GenBank databases. Due to the limitations of the sequence search tools, these services are mainly useful for the detection of closely related sequences. ReHAB[12] and Re-searcher[13] employ the more sensitive method PSI-BLAST[14], but they need to be installed, configured and maintained locally. DbW[15] aims to update user-supplied alignments with homologous and functionally related sequences, using the HMMer method[16] to search Swiss-Prot and TREMBL. Except for Re-searcher, these tools do not provide an option to choose the target database or search parameters, and none except Swiss-shop allows to change preferences later. Most importantly, none of these tools allows to search the PDB database and none makes use of the reliable and considerably more powerful profile-profile comparison tools.

PDBalert is a new web-based automatic system for protein homology detection, which checks the PDB database every week for templates homologous to the proteins in the users' watch lists. PDBalert performs searches with HHpred[17], a very sensitive and reliable remote homology detection server based on pairwise comparison of profile Hidden Markov models (HMMs)[18]. As soon as a homolog to a protein of interest is found in the PDB or among the sequences on-hold that will soon be released to the PDB, the user is notified with an email containing the link to the results page and to a 3D homology model.

## Methods

**Figure 1:**
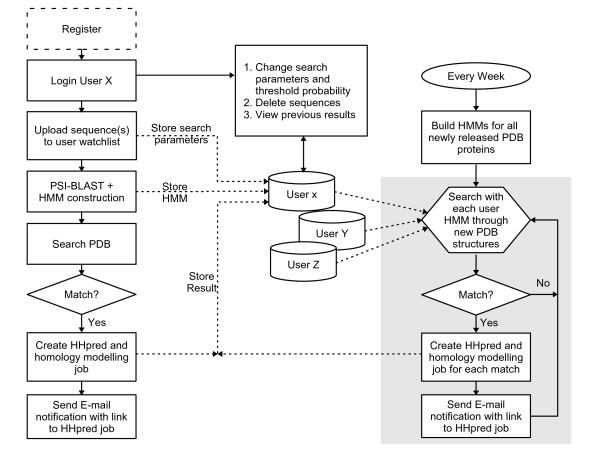
PDBalert flow chart (see Methods section).

**Figure 2:**
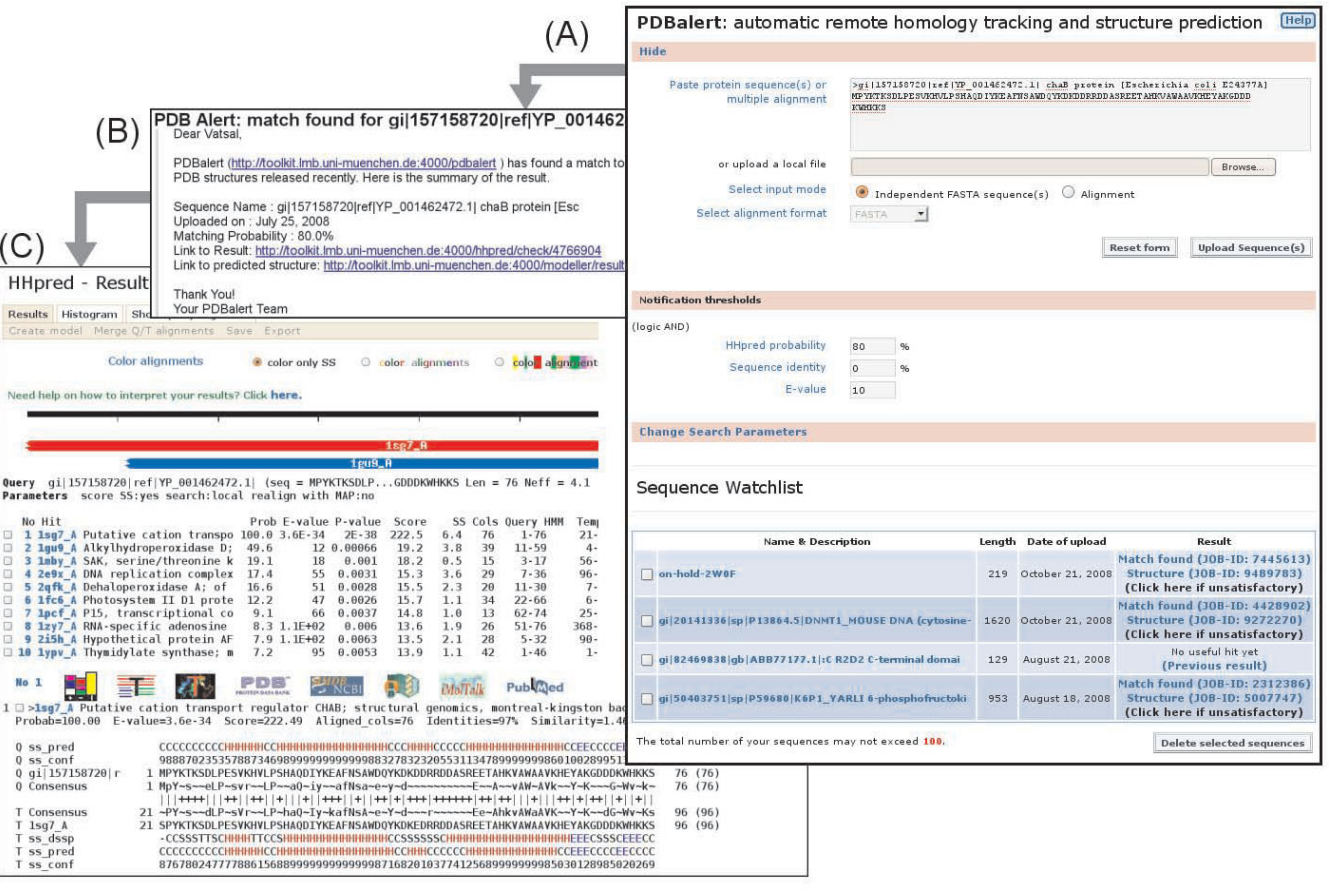
**Representative screen shots**. (A) PDBalert web interface with sequence upload section and personal watch list. (B) Email alert sent when significant hit is detected. (C) HHpred Results page containing alignment to PDBalert match.

The left part of the flow diagram in Fig. 1 illustrates the steps during and upon uploading of sequences to a user's "watch list", while the right part details the weekly procedure of checking for new hits among the newly released structures. After registering and logging in to the Bioinformatics Toolkit[19] (Fig. 1, left), users can upload protein sequences to their watch lists kept in their accounts (Fig. 2A for a screenshot). Input can be one or more independent FASTA sequences, or a multiple sequence alignment in one of ten common formats. Search parameters may be modified and are kept in a central MySQL database (Fig. 1, middle). Upon uploading a query sequence or alignment, an alignment of homologs is built by the buildali.pl script from the HHsearch package[18], which is also employed in HHpred. Next, a profile HMM is generated from the multiple alignment. The query HMM is then compared using HHsearch with HMMs representatives of all PDB structures and all sequences currently on hold (downloaded from ). Three thesholds can be specified by the user to decide when an e-mail notification should be sent (HHpred probability, sequence identity, E-value). If the query protein matches a protein in the PDB (or among the on-hold sequences) according to all three threshold criteria, the user will be notified with an e-mail (see Fig. 2B) containing a link to the results page and to a 3D homology model created by the MODELER package[20] using the HHpred alignment with the newly identified template (Fig. 2C). All results are also stored in the database for at least 6 months. They can be accessed via links in the user's watch list (Fig. 2A), which also allows to add or delete sequences and to change search parameters and threshold probabilities.

Every week, newly released PDB structures are obtained and an HMM for each of them is generated (Fig. 1, right). They are then compared with all sequences in the users' watch lists, and email notifications are sent to those users whose sequences get hits that meet the user-definable threshold criteria.

Whenever possible, users should upload sequences of single protein domains, since sensitivity increases and the false discovery is rate reduced compared to multiple domains. When PDBalert confidently predicts a domain in a longer sequence, it is therefore recommended to split the sequence at the boundaries of the discovered domain and upload the segments separately to PDBalert. In practice, it may be useful to leave some overlap of up to 30 residues between the segments when domain boundaries are not precisely known.

The web-interface of PDBalert is built on a Ruby on Rails[21] architecture on a Linux platform together with a MySQL[22] database for storing user inputs and preferences. Users do not require anything except a web-browser. PDBalert is integrated into the Bioinformatics Toolkit, a user-friendly web system of interlinked tools for protein sequence analysis and structure prediction.

## Discussion

The biannual CASP benchmarks[1] as well as the many studies employing state-of-the-art remote homology detection and structure prediction servers such as FFAS[23], HHpred[17], SAM-T2K[24], 3DJury[25], and I-TASSER[26] testify to the usefulness of these automatic methods. However, we believe that their full potential is far from being fully exploited. The principle reasons are that (1) innovations take time to spread; (2) most servers do not have user-friendly interfaces nor help pages; (3) Only few servers provide reliable significance estimates; (4) The servers are generally too slow to allow one to wait for the results on-line, taking hours or days to finish and discouraging usage on a regular basis. PDBalert addresses the last point in particular, by noting that most biologists and biochemists will have a fairly limited and conserved set of proteins in the focus of their attention. PDBalert saves these users the time to periodically redo searches for new templates to these proteins.

## Conclusion

The usefulness of PDBalert is owed to a large extent to the power of its underlying remote homology detection and structure prediction protocols, borrowed from HHpred. Two fully automated versions of HHpred that use the same homology detection method as PDBalert were ranked 2nd (HHpred2, multiple template modelling) and 8th (HHpred1, single template modelling, used by PDBalert to build a model with the detected template) out of a total of 68 automatic servers in the last community-wide protein structure prediction benchmark CASP7[1], while being more than 50 times faster than the other top servers. This speed allows to offer remote homology detection and structure prediction services for an automatic recurrent search to a wider community. We hope that PDBalert will encourage many more biologists to profit from recent advances in remote homology detection and structure prediction.

## Availability and requirements

• **Project name**: PDBalert

• **Project home page**: 

• **Operating system(s)**: Platform independent (web service)

• **Programming language**: Ruby

• **Licence**: None (Freely available to all academic and non-academic users)

## Authors' contributions

VA developed the PDBalert system, MR integrated the on-hold sequence database, AB, MR and JS coordinated the development and tested the application, JS conceived of the project, and VA and JS wrote the mansucript. All authors read and approved the final manuscript.
